# Transcatheter Closure of a Traumatic VSD with an ASD Occluder

**DOI:** 10.5935/abc.20180122

**Published:** 2018-08

**Authors:** Rui Alexandre Pontes dos Santos, Henrique Guedes, Leonor Marques, Carolina Lourenço, João Carlos Silva, Paula Pinto

**Affiliations:** 1 Centro Hospitalar do Tâmega e Sousa, Penafiel – Portugal; 2 Centro Hospitalar de São João, Porto – Portugal

**Keywords:** Heart Septal Defects, Ventricular / complications, Myocardial Contusions, Hemolysis, Heart Septal Defects, Ventricular / surgery

## Introduction

Traumatic ventricular septal defects (VSD) are exceptionally rare. They can be a consequence of either blunt or penetrating trauma. It is believed that most patients die before reaching the hospital, which makes this condition even more challenging.

Percutaneous closure of traumatic VSD has been presented as an alternative to open conventional surgery.^1^ Transcatheter intervention may have some benefits. The objective of this case report is to present a situation where the defect was closed using an Amplatzer septal occluder.

### Case Report

**Figure 1:**
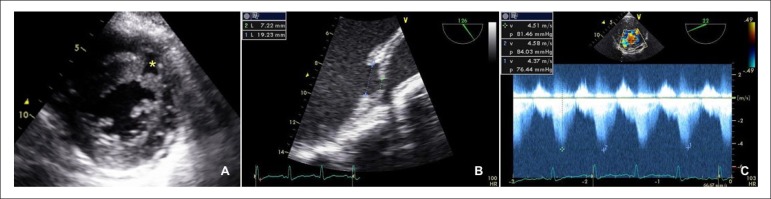
Echocardiographic images of the VSD. Panel A: VSD located in the mid anteroseptal segment. Panel B: VSD measuring 19 mm on the LV side and 7 mm on the RV side. Panel C: continuous wave Doppler estimating peak gradient at 84 mmHg.

A 23-year-old man was admitted in the emergency department after a frontal car collision. He had suffered severe blunt trauma, which included cervical subcutaneous emphysema, bilateral pulmonary contusion, left hemothorax, pneumomediastinum and complex fractures of both femurs. He was in hemorrhagic shock and was immediately taken to the operatory room. After external fixation of both femurs and reaching hemodynamic stability, he was transferred to the Intensive Care Unit. The following morning the presence of a loud holosystolic murmur was noted. The 12-lead electrocardiogram showed only sinus tachycardia. A transthoracic and later a transesophageal echocardiogram (TEE) were performed and both demonstrated a large muscular ventricular septal defect, located in the mid anteroseptal segment with signs of dissection through the basal septum (Figure 1). It measured 19 mm on the left ventricular (LV) side and 7 mm on the right ventricular (RV) side. The peak left to right shunt gradient was estimated in 84 mmHg and the Qp/Qs ratio was estimated in 1.8/1.0. Cardiac catheterization showed limited hemodynamic repercussion (systolic pulmonary artery pressure of 35 mmHg and a Qp/Qs ratio of 1.9/1.0) and the patient remained clinically stable, so a conservative strategy was decided at that time to allow the edges to heal and create a more delimited defect.

He was released after recovering from orthopedic surgery.

Three months later the patient was reevaluated and remained asymptomatic. He repeated cardiac catheterization, which showed a Qp/Qs ratio of 2.95/1.0. Because the shunt increased significantly, it was decided to close the defect percutaneously.

**Figure 2:**
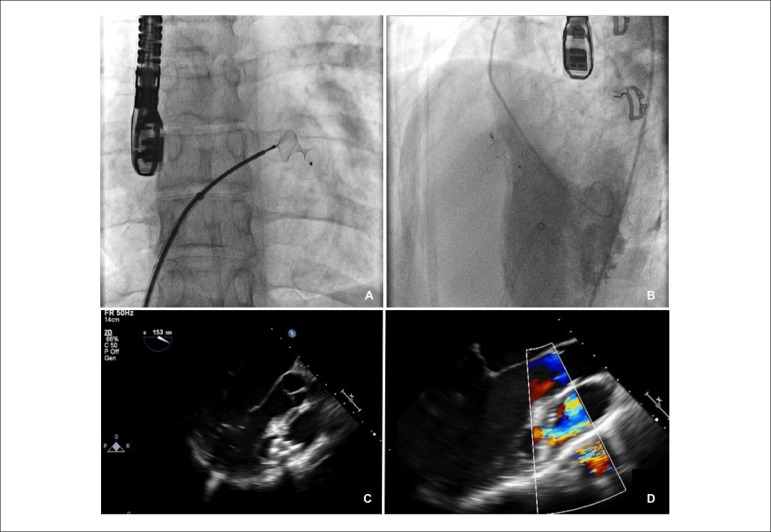
10 mm ASD Occluder deployed in the VSD. Panel A: position of the device in the VSD. Panel B: LV angiogram showing mild residual shunt. Panel C and D: TEE displaying the residual shunt through the superior edge of the device.

The procedure was done under general anesthesia and guided by transesophageal echocardiography. Cardiac catheterization was performed using the right femoral artery (6-Fr sheath) and vein (7-Fr sheath) and unfractionated heparin was administered. Angiogram of the LV confirmed a VSD with an oblique entry from the LV into the right ventricular outflow tract. The VSD was crossed using a retrograde arterial approach with a floppy guidewire, which was advanced into the pulmonary artery. The guidewire then snared and brought out the femoral venous sheath. This created an arteriovenous loop to allow the delivery of the closure device. A NuMed sizing balloon catheter was subsequently utilized to measure the defect, but it was not possible to maintain it steady. Therefore, the echocardiographic calculations were used to choose the device size. An 8-mm Amplatzer septal occluder was first selected and loaded into the sheath. The device was advanced across the VSD, but prolapsed back to the RV when it was being released. After this failed attempt, a slightly different approach was used. The VSD was crossed once more using the guidewire, this time in the opposite direction into the right subclavian artery. Once again, it was snared to make an arteriovenous loop, but on this occasion pulled out through the femoral arterial sheath. For this second attempt, it was decided to employ a 10-mm Amplatzer septal occluder. The device was advanced through the venous sheath and this time was successfully placed (Figure 2). LV angiogram after the procedure revealed a mild residual shunt and the Qp/Qs ratio reduced to 1.53/1.0.

A transesophageal echocardiography was repeated a month after the procedure, which showed the device well adapted to the defect. Nevertheless, a residual shunt remained in the superior border of the device with a peak gradient estimated in 90 mmHg (Figure 2).

Another complication of this procedure was the appearance of transient self-limited hemolysis. Initial blood analysis showed a LDH value > 2000 UI/L and haptoglobin < 6 mg/dL. The condition remained stable and resolved without the need of blood transfusions.

The patient continued to be asymptomatic and has returned to his previous professional life.

## Discussion

There are a few possible mechanisms that explain the development of traumatic VSDs. In this case, cardiac contusion after compression between the sternum and the spine or due to high intrathoracic pressures at impact seems to be the most probable explanation.^1^

During his stay our patient was clinically improving, which led to our decision to delay the intervention. Furthermore, it is known that in VSDs fibrotic tissue facilitates the device placement in elective closures.^2^ However, the progressively increasing shunt, led to the decision of closing it. He was initially considered for surgery, but given the risks associated this procedure, the alternative approach was pondered. Transcatheter closure can be a successful substitute with some advantages. It removes cardiopulmonary bypass, avoids arrhythmogenic scar formation related with ventriculotomy and reduces hospital stay and recovery time.

Because these are rare cases with diverse features, it can be challenging to size accurately the defect. In this case, imprecise echocardiographic measurements and the difficulty in operating the sizing balloon catheter, led to the inappropriate choice of the first device.

A possible complication of selecting this type of devices is the appearance of hemolysis. The probable mechanism is the passage of high-velocity turbulent blood flow through the device, which causes mechanical fragmentation of erythrocytes. Although there are reports of chronic hemolysis, it is usually self-resolving.^3^ Like previous cases,^4^^,^^5^ we encountered the same complication. Our patient remained asymptomatic and the hemolysis resolved without the need of blood transfusions.

## Conclusion

Transcatheter devices can be selected as the first choice for closing traumatic VSD. We demonstrate that ASD Occluder can be successfully implanted and that acceptable clinical effectiveness can be achieved.

## References

[1] Rollins MD, Koehler RP, Stevens MH, Walsh KJ, Doty DB, Price RS (2005). Traumatic ventricular septal defect: case report and review of
the English literature since 1970. J Trauma.

[2] Dehghani P, Ibrahim R, Collins N, Latter D, Cheema AN, Chisholm RJ (2009). Post-traumatic ventricular septal defects--review of the
literature and a novel technique for percutaneous closure. J Invasive Cardiol.

[3] Martinez MW, Mookadam M, Mookadam F (2007). A case of hemolysis after percutaneous ventricular septal defect
closure with a device. J Invasive Cardiol.

[4] Pesenti-Rossi D, Godart F, Dubar A, Rey C (2003). Transcatheter closure of traumatic ventricular septal defect: an
alternative to surgery. Chest.

[5] Suh WM, Kern MJ (2009). Transcatheter closure of a traumatic VSD in an adult requiring an
ASD occluder device. Catheter Cardiovasc Interv.

